# The cognitive neural mechanism of response inhibition and error processing to fearful expressions in adolescents with high reactive aggression

**DOI:** 10.3389/fpsyg.2022.984474

**Published:** 2023-01-04

**Authors:** Lijun Sun, Ziqi Liu, Yan Zhang, Yaopeng Jing, Yang Lei, Yuanyuan Zhang

**Affiliations:** ^1^School of Psychology, Xinxiang Medical University, Xinxiang, China; ^2^School of Psychology, Central China Normal University, Wuhan, China

**Keywords:** reactive aggression, adolescents, response inhibition, error processing, fearful expressions, Go/No-Go task

## Abstract

Reactive aggression in adolescents is characterized by high levels of impulsivity. This is associated with deficits in response inhibition and error processing and spontaneous emotion-driven responses to a perceived threat. However, the characteristics and cognitive neural mechanisms of response inhibition and error processing to indirect threat in adolescents with high levels of reactive aggression are unclear. This study explored the characteristics and cognitive neural mechanisms of response inhibition and error processing to fearful expressions in adolescents with high levels of reactive aggression using an emotional Go/No-Go paradigm combined with ERP recordings. Adolescents with high levels of reactive aggression (*n* = 31) and a control group (*n* = 30) took part in this study. Results showed that when presented with fearful expressions, adolescents with high levels of reactive aggression showed a smaller No-Go P3 effect and smaller ERN amplitudes following commission errors on the No-Go task than the control group. Results suggested that when presented with fearful expressions, adolescents with high levels of reactive aggression have impaired response inhibition in the later stage of actual inhibitory control of the motor system and impaired error processing in the early stage of fast and automatic initial error detection.

## 1. Introduction

Reactive aggression is caused by an individual’s perception of hostile provocation from others. It is a defensive response to threatening stimulation in the outside world, which is manifested as a loss of control. Adolescence is a sensitive period for the onset of reactive aggression in threatening situations (Jara et al., 2017). Reactive aggression can have serious negative impacts on the physical and mental health and the social adjustment of adolescents, causing further internalized problems, such as anxiety and depression, which in turn increase the risk of suicide (Hartley et al., 2018). Therefore, reactive aggression in adolescents remains an important focus of psychological and sociological research (McEwen and McEwen, 2017). Further study of reactive aggression and the mechanisms underpinning its development is of great significance.

Impulsivity is thought to be one of the underlying mechanisms of reactive aggression, which is defined as behavior without sufficient thought (Verdejo-García et al., 2008). Impulsivity is a multidimensional concept that includes personality, behavioral, and biological components. These dimensions can be measured using self-report measures (e.g., the impulsivity questionnaire), behavioral measures (e.g., response time and accuracy tasks), and imaging techniques (e.g., event-related potentials). In the behavioral dimension, impulsivity is used to describe maladaptive behaviors including deficits in response inhibition, i.e., the ability to inhibit inappropriate and unwanted behavior (Luijten et al., 2011), and deficits in error processing, i.e., the ability to monitor and evaluate ongoing behavior (Littel et al., 2012). It is proposed that impulsive behaviors, such as reactive aggression, deficits in response inhibition, and error processing, lead to the continuation of harmful behaviors despite awareness of negative consequences for the self and others (Luijten et al., 2011).

In the biological dimension of impulsivity, research has mainly focused on the brain activity accompanying maladaptive behaviors such as impaired response inhibition and error processing. A frequently used technique to measure these processes is the measurement of event-related potentials (ERPs). In the study of ERPs, the Go/No-Go paradigm is widely used to measure response inhibition as well as error processing because of its simple and clear cognitive components (Luijten et al., 2014). Two main event-related potential (ERP) components are demonstrated in Go/No-Go tasks related to response inhibition: the N2 and P3 components. The N2 is remarkably higher for No-Go compared to Go trials, with a frontally distributed negative waveform at 200–400 ms after the stimulus is presented, the so-called No-Go N2 effect. This is thought to reflect the early stage of an inhibitory process associated with conflict monitoring before a correct response (Donkers and van Boxtel, 2004). The P3 is remarkably higher for No-Go compared to Go trials, with a fronto-centrally distributed positive waveform at 300–700 ms after the stimulus is presented, the so-called No-Go P3 effect. The No-Go P3 effect reflects the later stage of actual inhibitory control of the motor system (Smith et al., 2008). Conflict monitoring in the early stage and inhibitory control in the late stage of response inhibition is impaired in adolescents with high levels of reactive aggression (Lievaart et al., 2016; Zhang et al., 2017; Hecht and Latzman, 2018). Response inhibition is an elastic resource that plays a role in specific contexts (Lievaart et al., 2018), and reactive aggression is a kind of impulse response to an externally threatening stimulus. It is therefore necessary to study the response inhibition to threatening situations in adolescents with high levels of reactive aggression. Our previous research found that when facing angry expressions, compared to the control group, adolescents with high levels of reactive aggression had the same No-Go N2 effect and a smaller No-Go P3 effect, indicating that response inhibition to angry expressions in adolescents with high levels of reactive aggression is impaired at the later stage of the actual inhibitory control (Sun et al., 2020a). In further research, other threatening expressions (such as fear) should be used to further explore the characteristics and cognitive neural mechanisms of response inhibition to threatening situations in adolescents with high levels of reactive aggression.

Two main ERP components are also demonstrated in Go/No-Go tasks related to error processing: error-related negativity (ERN) and error-related positivity (Pe). The ERN occurs within approximately 0–50 ms after an incorrect response and is maximal at fronto-central sites. It is thought to reflect fast and automatic initial error detection (Han and Jia, 2016). The ERN is always followed by the Pe, which emerges within approximately 200–600 ms after an incorrect response. It is maximal at parietocentral sites and is thought to be associated with a later stage of processing involving the conscious erroneous response or regulation of behavior (Luijten et al., 2014). Error-processing deficits are assumed to contribute to the continuation of reactive aggression (Luijten et al., 2011). Reduced Pe and intact ERN amplitudes have been frequently found in impulsive violent patient samples, such as those of violent offenders with psychopathy (Brazil et al., 2009), female incarcerated psychopaths (Maurer et al., 2015), and impulsive-violent offenders (Chen et al., 2014). Interestingly, ERN is related to the impulsive antisocial factor (Heritage and Benning, 2013). Importantly, Munro et al. (2007) adopted a standard letter flanker task and a face flanker task that needed discrimination between angry and fearful expressions and found that the ERN elicited by letter flanker errors was the same between groups but was significantly reduced in violent offenders during the face flanker task. Thus, it can be seen that the error processing mechanism between threatening and non-threatening situations differs in violent offenders compared to others. Adolescents with high levels of reactive aggression are often exposed to threatening situations (Wilkowski and Robinson, 2012), making it important to understand the characteristics of their error processing in threatening situations. One approach would be to study adolescents with high levels of reactive aggression within the normal population to further explore the characteristics and the underlying cognitive neural mechanisms of error processing in threatening situations.

Fearful expressions are often used in threatening situations. They are powerful social signals that alert the perceiver to the presence of a potential threat (Hortensius et al., 2016). Compared to angry expressions, which represent a direct threat, fearful expressions, as an indirect threat, are ambiguous in that they show the presence of danger but not its source (Hortensius et al., 2016). We explored the characteristics and underlying cognitive neural mechanisms of response inhibition and error processing to fearful expressions in adolescents with high levels of reactive aggression using an emotional Go/No-Go paradigm combined with ERP recordings.

In identifying the internal mechanism underlying reactive aggression in adolescents, we would be able to provide psychological interventions focusing on response inhibition and error processing to prevent adolescents from spiraling into violent crime. We also sought to provide a theoretical basis and objective biological markers for the establishment of a risk warning system and clinical intervention for adolescents with high levels of reactive aggression. The violence inhibition mechanism (VIM) is a neurobiological network thought to inhibit aggressive behavior through the elicitation of empathic response to the perception of distress in others. It comprises two stages: affect perception and motor extinction (involving response inhibition and error processing). Fearful expressions are one expression of distress affecting perception, the first stage of the VIM, which is impaired in adolescents with high levels of reactive aggression (Philipp-Wiegmann et al., 2017). This leads them to lose empathic responses to the perception of distress in others and a decrease in response inhibition and error processing. Thus, we expected that when presented with fearful expressions, adolescents with high levels of reactive aggression would show impaired response inhibition as reflected by smaller No-Go N2 effects, smaller No-Go P3 effects, and error processing as reflected by reduced ERN and Pe amplitude compared to the control group. At the behavioral level, we expected that when presented with fearful expressions, adolescents with high levels of reactive aggression would make more mistakes in the No-Go task.

## 2. Materials and methods

### 2.1. Participants

Using the effect sizes of group differences between the aggressive and control groups in existing studies of emotional response inhibition (η*_p_*^2^ = 0.29; Sun et al., 2020a) and error processing (η*_p_*^2^ = 0.35; Munro et al., 2007), we adopted G*Power 3.1 software (with power set to 95% and alpha level set to 0.05) and calculated the sample size in each group to be 14 and 15, respectively. We obtained the written informed consent of participants and permission of the local Ethics Committee. In total, 1,200 freshmen (of whom 600 were males) from a Chinese public university were recruited. We selected participants based on responses to the Chinese version of the Aggression Questionnaire (AQ) and the Reactive-Proactive Aggression Questionnaire (RPQ). Participants with BPAQ and reactive aggression scores greater than mean + 1σ and the difference between reactive aggression and proactive aggression in the RPQ being greater than mean + 1σ comprised the high reactive aggression group. The control group comprised participants with lower BPAQ and reactive aggression scores (< mean + 0.5 SD). We also randomly selected 31 participants (15 male subjects) from the high reactive aggression group and 30 participants from the control group (15 male subjects). The age range of the sample was 17–19 years. All participants had a normal or corrected-to-normal vision and were right-handed. There was no significant difference in age between the group with high reactive aggression (*M* = 18.35, *SD* = 0.84) and the control group [*M* = 18.34, *SD* = 0.79; *t*(59) = 0.05, *p* = 0.96]. The scores for reactive aggression in the high reactive aggression group (*M* = 12.94, *SD* = 1.24) were significantly higher than that in the control group [*M* = 5.05, *SD* = 1.46; *t*(59) = 23.06, *p* < 0.001].

### 2.2. Questionnaires

The Chinese version of the AQ (Buss and Perry, 1992; Luo, 2008) was used to measure aggressive behavior. It consists of 29 items that can be divided into four dimensions: physical aggression, verbal aggression, hostility, and anger. Participants were requested to respond on a five-point Likert scale (1 = “extremely uncharacteristic of me,” 5 = “extremely characteristic of me”). A higher score indicates higher levels of aggressive behavior. In this study, the AQ exhibited good internal reliability (α = 0.88).

The Chinese version of the RPQ (Raine et al., 2006; Zhang et al., 2014) was used to measure proactive and reactive aggression. It consists of 23 items that can be divided into two dimensions: reactive aggression (11 items) and proactive aggression (12 items). Participants were requested to respond on a three-point Likert scale (0 = “never,” 1 = “sometimes,” 2 = “often”). In this study, the proactive aggression and reactive aggression subscales showed good internal reliability (α = 0.89 and α = 0.85).

### 2.3. Emotional face stimuli

The experimental materials consisted of 54 images of angry faces and 54 images of fearful faces selected from the Chinese Facial Affective Picture System (Wang and Luo, 2005). There was no significant difference in intensity between angry pictures and fearful pictures (*t* = −1.79, *p* > 0.05; angry: 5.75 ± 1.10, fearful: 6.11 ± 1.02). Both picture types were matched for gender, color, size, and contrast.

### 2.4. Emotional Go/No-Go task

**FIGURE 1:**
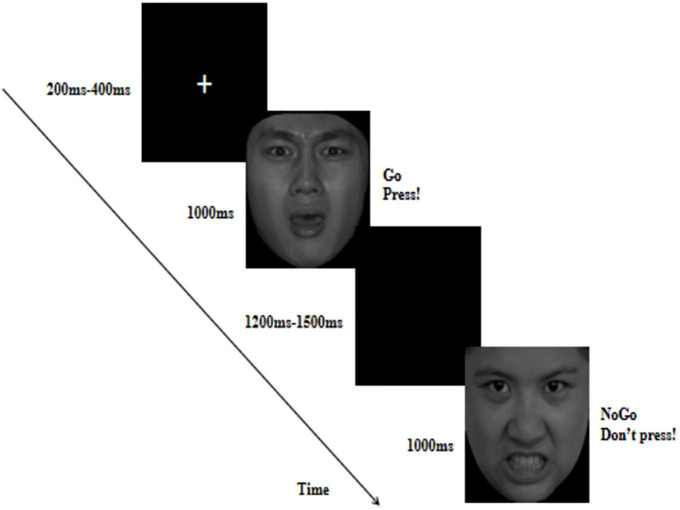
The flow map of emotional Go/No-Go task in which fearful faces served as Go cues and angry faces served as No-Go cues. Fearful and angry facial images reproduced with permission from Chinese Facial Affective Picture System (Wang and Luo, 2005).

An emotional Go/No-Go task was used to measure response inhibition and error processing in response to fearful expressions. Existing research has indicated that distressed and angry facial stimuli can best reflect VIM (Fido et al., 2017). Meanwhile, individuals with high levels of reactive aggression are often exposed to threatening situations (Wilkowski and Robinson, 2012). Therefore, in this task, we used angry expressions, which is a direct threat, as a control. Angry and fearful pictures were used as frequent Go or infrequent No-Go stimuli. In each trial, a fixation point was presented for 200–400 ms, followed by an angry or fearful picture for 1,000 ms, and then a black screen for 1,200–1,500 ms as shown in Figure 1. Participants were instructed to respond as quickly and accurately as possible to the Go stimuli by pressing a button with their index fingers and to withhold their response to the No-Go stimuli. The task was divided into two blocks: anger Go/fear No-Go and fear Go/anger No-Go. Each block consisted of 180 trials, of which 30% were No-Go and 70% were Go trials, resulting in 126 Go trials and 54 No-Go trials. Go trials always preceded No-Go trials to induce pre-potent conflict and motor responses during response inhibition. The order of the two blocks was balanced between the subjects. Each participant completed 360 trials. Participants performed two short practice blocks before starting the actual experiment. Between the two blocks, participants could take a short break.

### 2.5. Electrophysiological recording and analysis

The electroencephalographic (EEG) data were recorded using the 64-channel Neuroscan system (Neuroscan SynAmps2; NeuroScan Inc., Sterling, VA, United States) according to the extended international 10/20 system. Electrode impedance was kept below 5 kΩ. All signals were digitized with a sampling rate of 500 Hz and 32-bit A/D conversion with a band-pass filter of 0.05–100 Hz.

The electroencephalographic data were offline-referenced to the average of the left and right mastoids. A filter with a bandpass of 0.15–30 Hz was used to remove high-frequency noise. Independent component analysis (ICA) was used to reject the blinks and eye movement artifacts. In this step, we first performed ICA and then rejected components by map. We recognized and removed blink and eye drift components according to the component distribution in the forehead: relatively front, random distribution, and low-frequency with high energy. Epochs with a voltage exceeding ± 75 μV were excluded. EEG data were segmented into epochs from 200 ms before stimulus onset to 1,000 ms after stimulus onset and from 200 ms before the response onset to 800 ms after the response onset. The mean 200-ms pre-stimulus or pre-response period served as a baseline.

For the N2 and P3 components, the base-peak ERP amplitudes were calculated. Segments with incorrect responses (misses for Go trials or false alarms for No-Go trials) were eliminated from the analyses. The N2 and P3 were defined as the peak amplitude within the 210–420 ms and 350–750 ms range after the stimulus was present and were studied at F3, Fz, F4, FC3, FCz, and FC4 and FC3, FCz, FC4, C3, Cz, and C4, respectively (Euser and Franken, 2012; Sun et al., 2020a). The average number of available Go and No-Go trials for the N2 and P3 components was 100 and 40 for fearful pictures and 80 and 36 for angry pictures. Overall, 20.63 and 25.93% of epochs were rejected for Go and No-Go trials in the fear condition, and 36.51 and 33.33% of epochs were rejected for Go and No-Go trials in the anger condition. Four participants in total (two adolescents with high levels of reactive aggression and two control adolescents) were excluded from ERP analyses because there were fewer than 20 artifact-free N2 or P3 epochs (Rietdijk et al., 2014). Data from 29 adolescents with high levels of reactive aggression and 28 control adolescents were included in the statistical analysis. There was no significant difference in the epoch percentage of rejection between adolescents with high levels of reactive aggression and control adolescents for Go and No-Go trials in the fear or anger condition (*p*-values > 0.05).

For the ERN and Pe components, the base-peak ERP amplitudes were calculated for incorrect No-Go trials. The ERN and Pe were defined as the peak amplitude within the 0–50 ms and 100–500 ms ranges after the response was present and were studied at FZ, FCz, CZ, and CPz and FCz, CZ, CPz, and Pz, respectively (Littel et al., 2012). The average number of available incorrect No-Go trials for the ERN and Pe components was 12 for fearful pictures and 16 for angry pictures. A total of 10 participants (four adolescents with high reactive aggression and six control adolescents) were excluded from ERP analyses because there were less than six artifact-free ERN or Pe epochs in at least one of the experimental conditions (Lievaart et al., 2016). Data from 27 adolescents with high levels of reactive aggression and 24 control adolescents were included in the statistical analysis.

### 2.6. Statistical analyses

#### 2.6.1. Behavioral analysis

A 2 (Group: high reactive aggression, control) × 2 (Emotion: fearful expressions, angry expressions) × 2 (Inhibition: Go, No-Go) repeated-measures ANOVA was conducted to analyze group differences regarding the behavioral error rates, and a 2 (Group: high reactive aggression, control) × 2 (Emotion: fearful expressions, angry expressions) repeated-measures ANOVA was performed to analyze group differences with respect to the reaction times on correct Go trials. Response times outside of 150–1,500 ms were excluded.

#### 2.6.2. ERP analyses

A Group (high reactive aggression group, control group) × Emotion (fearful expressions, angry expressions) × Inhibition (Go, No-Go) × Electrode (F3, Fz, F4, FC3, FCz, and FC4 for N2 and FC3, FCz, FC4, C3, Cz, and C4 for P3) repeated-measures ANOVA was performed for the stimulus-locked N2 and P3 amplitudes. To analyze the No-Go N2 effect and No-Go P3 effect more clearly, the Go amplitude was subtracted directly from the No-Go amplitudes on each emotion condition at each electrode. A Group (high reactive aggression group, control group) × Emotion (fearful expressions, angry expressions) × Electrode (F3, Fz, F4, FC3, FCz, and FC4 for N2 and FC3, FCz, FC4, C3, Cz, and C4 for P3) repeated-measures ANOVA was performed for the stimulus-locked N2 and P3 difference waves. A Group (high reactive aggression group, control group) × Emotion (fearful expressions, angry expressions) × Electrode (FZ, FCz, CZ, and CPz for ERN and FCz, CZ, CPz, and Pz for Pe) repeated-measures ANOVA was carried out for the response-locked ERN and Pe amplitudes with a group as a between-subject factor and emotion and electrode as within-subject factors. Multiple comparisons for all significant ANOVA effects were further analyzed using Bonferroni-corrected *post hoc t*-tests. Simple effect analyses were conducted only for interactions that included the between-subject factor group.

## 3. Results

### 3.1. Behavioral results

#### 3.1.1. Error rates

**FIGURE 2:**
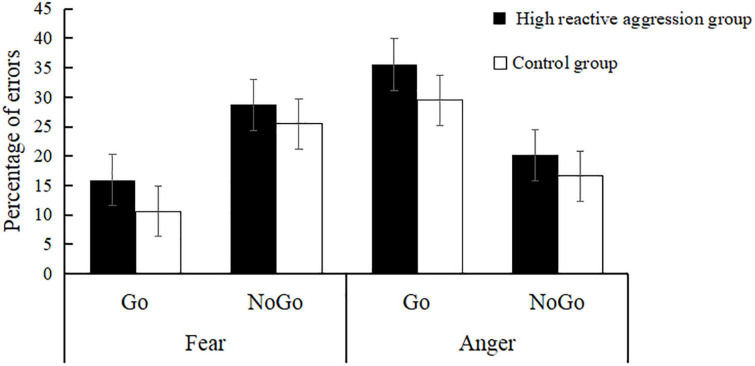
Error rates during the emotional Go/No-Go task by group.

A robust main effect was found for emotion [*F*(1,55) = 21.27, *p* < 0.001, η^2^ = 0.28] (see Table 1 and Figure 2), indicating that the error rate in the anger condition (25.45% ± 1.33%) was significantly higher than that in the fear condition (20.18% ± 1.22%).

**TABLE 1:** Behavioral results for the emotional Go/No-Go task by the group.

Group	Variable (unit)	Emotion			
		Fear		Anger	
		*M*	*SD*	*M*	*SD*
High reactive aggression group	OE (%)	15.93	1.86	35.55	3.42
	CE (%)	28.67	2.81	20.18	2.96
	Go RT (ms)	518.16	11.90	584.85	12.94
Control group	OE (%)	10.63	1.89	29.48	3.48
	CE (%)	25.53	2.86	16.60	3.01
	Go RT (ms)	588.45	12.12	546.63	13.17

CE, percentage of commission errors; OE, percentage of omission errors; RT, reaction time for correct Go trials; ms, milliseconds.

The interaction between emotion and inhibition was significant [*F*(1,55) = 70.79, *p* < 0.001, η^2^ = 0.56]. The simple effect analysis showed that for fearful expressions, the error rate of No-Go (27.10% ± 2.00%) was significantly higher for Go [13.27% ± 1.32%; *F*(1,55) = 33.91, *p* < 0.001]. For angry expressions, the error rate of Go (32.51% ± 2.43%) was significantly higher than that for No-Go [18.39% ± 2.11%; *F*(1,55) = 14.65, *p* < 0.001].

#### 3.1.2. Reaction time

The results showed that the main effects of group and emotion were not significant (see Table 1) in terms of reaction time.

There was a significant interaction effect for group and emotion (*F* = 14.02, *p* < 0.001, η^2^ = 0.42). Simple effect analysis showed that for angry expressions, there was no significant difference between the high reactive aggression group (584.84 ± 12.93 ms) and control group [546.63 ± 13.16 ms; *F*(1,55) = 2.37, *p* = 0.08]. For fearful expressions, reaction time in the control group (588.44 ± 12.11 ms) was significantly higher than the high reactive aggression group [518.15 ± 11.90 ms; *F*(1,55) = 5.67, *p* = 0.02].

### 3.2. N2 amplitudes

As shown in Table 2, the main effect of inhibition was significant [*F*(1,55) = 5.32, *p* = 0.025, η^2^ = 0.09], indicating that the amplitude of No-Go (−0.04 ± 0.56 μV) was significantly greater than that of Go (0.50 ± 0.55 μV). The main effect of the electrode was significant [*F*(5,275) = 7.68, *p* < 0.001, η^2^ = 0.12]. The amplitude at F4 (0.68 ± 0.50 μV) was significantly higher than that at FC3 (0.70 ± 0.52 μV, *p* = 0.004). The amplitude at Fz (−0.12 ± 0.63 μV) was significantly higher than that at FC3 (0.70 ± 0.52 μV, *p* = 0.002) and FC4 (0.68 ± 0.50 μV, *p* = 0.002). The amplitude at F3 (0.13 ± 0.55 μV) was significantly higher than that at FC3 (0.70 ± 0.52 μV, *p* = 0.004).

**TABLE 2:** N2 and P3 amplitudes during the emotional Go/No-Go task by the group.

Emotion	Inhibition	High reactive aggression group (*n* = 29)				Control group (*n* = 28)			
		N2		P3		N2		P3	
		*M*	*SD*	*M*	*SD*	*M*	*SD*	*M*	*SD*
Anger	Go	−0.48	0.80	9.14	0.75	0.75	0.81	11.04	0.77
	No-Go	−0.33	0.82	10.46	1.04	0.70	0.84	11.48	1.06
Fear	Go	0.13	0.81	9.95	0.91	1.62	0.83	11.94	0.92
	No-Go	−1.10	0.81	10.01	0.97	0.54	0.82	13.39	0.98

N2 and P3 are mean amplitudes in microvolts averaged across six recorded fronto-central scalp sites (F3, Fz, F4, FC3, FCz, and FC4 for N2 and FC3, FCz, FC4, C3, Cz, and C4 for P3).

The main effect of group and group-dependent interaction effects did not reach statistical significance (*p*-values > 0.05).

### 3.3. P3 amplitudes

As shown in Table 2, the main effect of emotion was significant [*F*(1,55) = 8.17, *p* = 0.006, η^2^ = 0.13], indicating that the amplitude of P3 in the fear condition (11.32 ± 0.64 μV) was significantly higher than that in the anger condition (10.53 ± 0.60 μV). The main effect of Go/No-Go was significant [*F*(1,55) = 5.50, *p* = 0.023, η^2^ = 0.09], showing that the No-Go amplitude (11.33 ± 0.68 μV) was significantly higher than the Go amplitude (10.52 ± 0.57 μV). The main effect of the electrode was significant [*F*(5,275) = 17.49, *p* < 0.001, η^2^ = 0.63], showing that the amplitude at C_*Z*_ (12.72 ± 0.75 μV) was significantly higher than that at FC3 (9.69 ± 0.58 μV), FC_*Z*_ (11.40 ± 0.68 μV), FC4 (10.36 ± 0.58 μV), C3 (10.18 ± 0.57 μV), and C4 (11.20 ± 0.58 μV; all *p*-values < 0.01).

There was a significant Group × Emotion interaction effect [*F*(1,55) = 4.82, *p* = 0.032, η^2^ = 0.01]. Simple effect analysis demonstrated that in the anger condition, the amplitude of the control group (11.26 ± 0.86 μV) was significantly higher than that of the reactive aggression group (9.80 ± 0.84 μV) [*F*(1,55) = 13.56, *p* < 0.001], and in the fear condition, the amplitude of the control group (12.66 ± 0.91 μV) was significantly higher than that of the high reactive aggression group (9.98 ± 0.90 μV) [*F*(1,55) = 3.24, *p* = 0.009]. The interaction between group, emotion, and electrode was significant [*F*(5,275) = 2.48, *p* = 0.032, η^2^ = 0.04]. The simple effect analysis showed that in the fear condition, at FC3, FC_*Z*_, FC4, and C4, the amplitude in the control group was significantly higher than that in the high reactive aggression group (*p*-values < 0.05).

Most interestingly, there was a significant Group × Emotion × Inhibition interaction effect [*F*(1,55) = 4.40, *p* = 0.041, η^2^ = 0.07]. The simple effect analysis showed that in the anger condition, the Go and No-Go amplitudes of the control group were the same as those of the high reactive aggression group (*p*-values > 0.05). In the fear condition, there was no significant difference in Go amplitude between the high reactive aggression group (9.95 ± 0.91 μV) and the control group [11.94 ± 0.92 μV; *F*(1,55) = 2.32, *p* = 0.134], and there was a significant group difference in No-Go amplitude. The No-Go amplitude of the control group (13.39 ± 0.98 μV) was significantly higher than that of the high reactive aggression group [10.01 ± 0.97 μV; *F*(1,55) = 6.02, *p* = 0.017].

### 3.4. N2 difference wave

The main effects of emotion, group, and the interaction effects for Group × Emotion, Group × Electrode, and Group × Emotion × Electrode did not reach statistical significance (*p*-values > 0.05).

### 3.5. P3 difference wave

**FIGURE 3:**
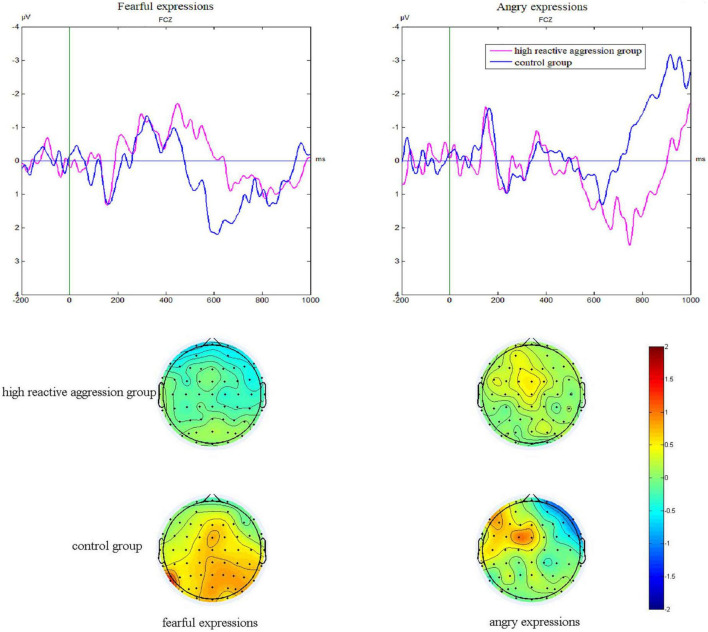
P3 difference waves of No-Go subtracted from Go trials at the FCz site and corresponding scalp topography at 580–650 ms of the two groups in fear and anger conditions.

As Figure 3 shows, the main effect of the electrode [*F*(5,275) = 9.82, *p* = 0.001, η^2^ = 0.07] was significant. The difference wave at FC_*Z*_ (1.33 ± 0.39 μV) was significantly higher than that at FC3 (0.64 ± 0.32 μV, *p* = 0.004) and C_*Z*_ (0.64 ± 0.36 μV, *p* = 0.035).

The Group × Emotion interaction was significant [*F*(1,55) = 4.40, *p* = 0.041, η^2^ = 0.07]. The simple effect analysis showed that in the fear condition, the difference wave (1.45 ± 0.55 μV) in the control group was marginally significantly higher than that in the high reactive aggression group (0.05 ± 0.54 μV; *F* = 3.27, *p* = 0.076), whereas in the anger condition, there was no significant difference between the control group (0.43 ± 0.69 μV) and the high reactive aggression group (1.32 ± 0.68 μV; *F* = 0.82, *p* = 0.369).

### 3.6. ERN amplitudes

As shown in Table 3 and Figure 3, we found the main effect of emotion [*F*(1,49) = 8.34, *p* = 0.006, η^2^ = 0.15]. The ERN amplitude after fearful expressions (−4.30 ± 0.59 μV) was significantly greater than anger expressions (−1.99 ± 0.65 μV). There was a main effect of the electrode [*F*(3,147) = 14.89, *p* < 0.001, η^2^ = 0.20] and the amplitude at F_*Z*_ was significantly greater than at other electrode points (*p*-values < 0.05).

**TABLE 3:** Error-related negativity and error-related positivity amplitudes during the emotional Go/No-Go task by the group.

Emotion	High reactive aggression group (*n* = 27)				Control group (*n* = 24)			
	ERN		Pe		ERN		Pe	
	*M*	*SD*	*M*	*SD*	*M*	*SD*	*M*	*SD*
Anger	−0.86	0.90	11.51	2.06	−3.12	0.95	13.92	2.18
Fear	−3.51	0.81	13.97	1.85	−5.09	0.85	15.23	1.97

ERN, error-related negativity; Pe, error-related positivity. ERN and Pe are mean amplitudes in microvolts averaged across four recorded fronto-central scalp sites and four recorded parietocentral scalp sites (FZ, FCz, CZ, and CPz for ERN and FCz, CZ, CPz, and Pz for Pe).

The main effect was found for the group [*F*(1,49) = 4.06, *p* = 0.047, η^2^ = 0.08], showing that the ERN amplitude of the high reactive aggression group (−2.19 ± 0.65 μV) was significantly lower than the control group (−4.11 ± 0.69 μV). The interactions between Group × Emotion, Group × Electrode, and Group × Emotion × Electrode were not significant (*p*-values > 0.05).

### 3.7. Pe amplitudes

**FIGURE 4:**
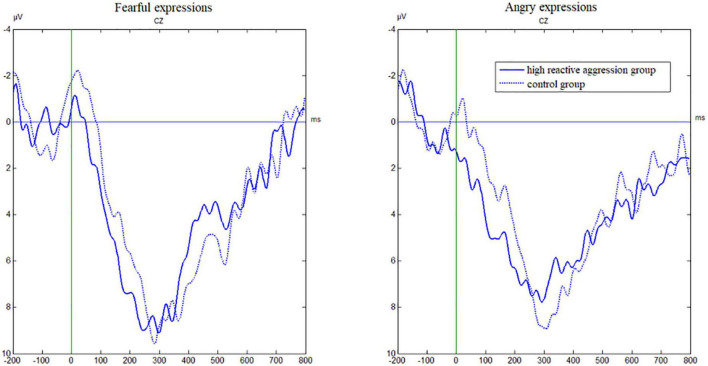
Grand average waveforms from Cz evoked by the emotion for incorrect No-Go trials in the emotional Go/No-Go task as a function of group.

As shown in Table 3 and Figure 4, the main effect of the electrode [*F*(3,147) = 101.32, *p* < 0.001, η^2^ = 0.67] was significant, and amplitude at F_Z_ was significantly higher than that at other electrode points (*p*-values < 0.001).

The main effect of the group was not significant [*F*(1,49) = 0.53, *p* = 0.471, η^2^ = 0.01]. The interaction between Group × Electrode was significant [*F*(3,147) = 3.69, *p* = 0.013, η^2^ = 0.07]. The simple effect analysis showed that the effect of the group was not significant at each electrode point (*p*-values > 0.05). There was no significant interaction between Group × Emotion and Group × Emotion × Electrode (*p*-values > 0.05).

## 4. Discussion

The main goal of this study was to investigate the characteristics and underlying cognitive-neural mechanisms of response inhibition and error processing to fearful expressions in adolescents with high levels of reactive aggression. Results showed that, as a whole, adolescents showed larger N2 and P3 amplitudes for the No-Go task compared to the Go task, indicating that the affective Go/No-Go task was valid in allowing participants to form a dominant response that is difficult to inhibit.

On the behavioral level, we found that when presented with fearful expressions, adolescents with high reactive aggression showed the same commission errors on the No-Go trials as the control group. This may be due to individuals overall being more sensitive to threatening expressions (Skinner et al., 2018), and as a result, the experimental task using fearful and angry expressions was easier, and individuals with high and low reactive aggression all demonstrated the same higher accuracy. It should be noted that there were trends in the data, such that when presented with fearful expressions, individuals with high reactive aggression made more commission errors relative to the control group. Meanwhile, the interaction between emotion and inhibition as seen in error rates implies that participants are generally more likely to avoid angry faces (errors go > No-Go) and more likely to approach fearful faces (error rate No-Go > Go). In addition, we found that when presented with fearful expressions, the reaction time in the high reactive aggression group was significantly shorter than in the control group, which could reflect adolescents with high levels of reactive aggression being less distracted by the implied threat or distress associated with fearful faces.

Regarding response inhibition, the ERP analyses showed that when presented with fearful expressions, adolescents with high levels of reactive aggression had a smaller No-Go P3 effect than the control group and the same No-Go N2 effect as the control group, which was partly consistent with our expectations. These results suggest that response inhibition to fearful expressions is impaired in adolescents with high reactive aggression, and this impairment occurs in the late stage of response inhibition, which is closely related to the actual inhibition of the motor system, rather than the early stage. The Integrative Cognitive Model (ICM) of reactive aggression (Wilkowski and Robinson, 2010) assumes an internal cognitive mechanism that functions in threatening situations, leading to reactive aggression. This model holds that when facing threatening situations, individuals will automatically interpret hostility, which leads to a decrease in response inhibition and thus reactive aggression. Based on anger expressions presenting a direct threat situation (Sun et al., 2020a), we used fear as an indirect threat expression to verify the ICM of reactive aggression from another perspective. Our previous research found that when faced with fearful expressions, individuals with high-trait aggression showed a smaller No-Go P3 effect and the same No-Go N2 effect compared to individuals with low-trait aggression (Sun et al., 2020b). Reactive aggression is a type of trait aggression. Our study focused on adolescents with high levels of reactive aggression and thus expanded the existing research on trait aggression.

Regarding error processing, the ERP analyses showed that when facing fearful expressions, the ERN amplitudes following commission errors on the No-Go task in adolescents with high reactive aggression were reduced compared to the control group, whereas the Pe amplitudes were comparable for both groups. This is consistent with existing research showing that the ERN is significantly reduced in violent offenders, while no group effect has been found on the Pe component in an emotional flanker task that needed individuals to discriminate between angry and fearful expressions (Munro et al., 2007). This suggests that when facing fearful expressions, error processing in adolescents with high reactive aggression is impaired, and this impairment likely occurs in the early stage reflecting fast and automatic initial error detection, rather than the late stage of error processing. Our results provide further support for the dissociation between early unconscious components of error processing and later conscious components leading to adaptive behavior. Studies have previously found that individuals with high aggression have poor performance in emotion recognition (Philipp-Wiegmann et al., 2017). Thus, affect perception, which is the first stage of the VIM, is impaired in individuals with high reactive aggression, which makes them less likely to experience an empathic response to the perception of fear in others, which leads to a decrease in response to inhibition and error processing. Meanwhile, according to the dual competition model (Pessoa, 2009), cognitive resources are finite, and as such, the results of this study may be attributable to individuals with high reactive aggression having deficits in fearful expression recognition (Philipp-Wiegmann et al., 2017) and thus requiring more cognitive resources, which in turn impairs subsequent response inhibition and error processing.

Interestingly, we found that when facing fearful expressions, there was a dissociation in the ERN and N2 results for adolescents with high reactive aggression. This is not consistent with related research, which has found that the N2 and ERN share the same scalp distribution and the same origin of the anterior cingulate cortex (ACC) (Yang et al., 2010). In response to inhibition tasks, the ACC plays a major role in conflict monitoring and error monitoring (Garavan et al., 2002). In the emotional response inhibition task, the ACC not only participates in response inhibition (Schulz et al., 2009) but also in emotional processing (Hare et al., 2008). More importantly, the ACC is involved in the interaction between response inhibition and emotional processing (Goldstein et al., 2007). Therefore, these inconsistent results may be due to the moderating effects of emotional tasks. Existing studies have shown a similarity in the neural sources that underlie the Pe and P3 components (Arbel and Donchin, 2009, 2010), and both are involved in the conscious processing of motivationally salient events (Ridderinkhof et al., 2009). Findings from our study using fearful expressions as background suggest a non-affected Pe but an impaired P3 effect in adolescents with high reactive aggression, suggesting that the two components are not completely equivalent. This is in accordance with existing research, which has used a non-emotional flanker-stop-signal task with juvenile violent offenders (Vilà-Balló et al., 2014).

In addition, the ERP analyses showed that when faced with angry expressions, adolescents with high reactive aggression showed the same No-Go P3 as the control group, which is inconsistent with our previous results using happy expressions as the control (Sun et al., 2020a). This inconsistency may be caused by differences in the control of emotions and response inhibition to angry expressions in adolescents with high reactive aggression may have situational particularity. Hence, an interesting avenue for future research would be to test the boundary conditions regarding the relationship between response inhibition to angry expressions and reactive aggression in adolescents. The ERP analyses also showed that the ERN amplitudes following commission errors on the No-Go task in adolescents with high reactive aggression were reduced compared to the control group, whereas the Pe amplitudes were comparable for both groups. This suggests that when faced with threatening expressions, error processing in adolescents with high reactive aggression is impaired.

There were some limitations to our study. First, we cannot draw conclusions regarding causality. While it might be that when facing fearful expressions, reduced response inhibition and poor error processing underlie the pathological causes of adolescent reactive aggression, it cannot be ruled out that reduced response inhibition and poor error processing are the results of reactive aggression. Second, only one expression (anger) was used as a control condition. Future studies should use a range of additional control expressions, including positive and neutral expressions, to explore the situational particularity of impaired response inhibition to fearful expressions in adolescents with high reactive aggression. Third, we recruited only freshmen as a representative group of adolescents. Further research should include students in middle school to increase the potential generalization of the current research results. Additionally, we only controlled for major extraneous variables such as age, sex, vision, and being right-handed. It would be useful to measure additional socio-demographic and psychological variables of the participants. Despite these limitations, the present findings are of great significance for understanding the internal mechanism of reactive aggression. Furthermore, specifically tailored interventions for reaction aggression in adolescents can now be proposed.

## 5. Conclusion

Our results show that response inhibition to fearful expressions in adolescents with high reactive aggression is impaired in the later stage of inhibitory control of the motor system in the pre-motor cortex, and error processing to fearful expressions in adolescents with high reactive aggression is impaired in the early stage of fast and automatic initial error detection.

## Data availability statement

The raw data supporting the conclusions of this article will be made available by the authors, without undue reservation.

## Ethics statement

The studies involving human participants were reviewed and approved by the Institutional Review Board of Xinxiang Medical University. Written informed consent to participate in this study was provided by the participants’ legal guardian/next of kin.

## Author contributions

LS: conceptualization, data curation, investigation, and methodology. YJ: formal analysis. ZL: resources. LS and YuZ: supervision. YL: writing — original draft. LS, ZL, and YaZ: writing — review and editing. All authors contributed to the article and approved the submitted version.
